# Real‐World Experience of Efficacy and Tolerability of Continuous Infusion Ifosfamide for Advanced Soft Tissue and Bone Sarcoma Patients: A Single Centre Retrospective Cohort

**DOI:** 10.1002/cam4.71614

**Published:** 2026-02-15

**Authors:** Ilaria Mascagni, Alice Laffi, Maria Susanna Grimaudo, Ferdinando Carlo Maria Cananzi, Laura Samà, Salvatore Lorenzo Renne, Federico D’Orazio, Pierina Navarria, Elena Clerici, Carlo Carnaghi, Sara Farinatti, Erika Stucchi, Marialudovica Le Moli, Laura Giordano, Robert G. Maki, Armando Santoro, Alexia Francesca Bertuzzi

**Affiliations:** ^1^ Department of Biomedical Sciences Humanitas University Pieve Emanuele Milan Italy; ^2^ Medical Oncology and Hematology Unit IRCCS Humanitas Research Hospital Rozzano Milan Italy; ^3^ Division of Medical Oncology Humanitas Gavazzeni Bergamo Italy; ^4^ Sarcoma, Melanoma and Rare Tumor Surgery Unit IRCCS Humanitas Research Hospital Rozzano Milan Italy; ^5^ Department of Pathology IRCCS Humanitas Research Hospital Rozzano Milan Italy; ^6^ Diagnostic Radiology Department IRCCS Humanitas Research Hospital Rozzano Milan Italy; ^7^ Radiation Oncology Unit IRCCS Humanitas Research Hospital Rozzano Milan Italy; ^8^ Oncology Department Humanitas Catania Catania Italy; ^9^ Biostatistic Unit, Humanitas Cancer Center IRCCS Humanitas Research Hospital Rozzano Milan Italy; ^10^ Memorial Sloan‐Kettering Cancer Center New York New York USA; ^11^ School of Medical Sciences Weill‐Cornell Medical College New York New York USA

**Keywords:** advanced sarcoma, bone sarcoma, continuous infusion ifosfamide, real‐world data, soft tissue sarcoma

## Abstract

**Background:**

In the absence of randomized trials, selecting second or later‐line therapies in advanced sarcoma generally hinges on histology, patient performance status, and toxicity profile. We herein report a real‐world sarcoma referral center experience on continuous infusion Ifosfamide (ciIFO), in advanced Soft tissue sarcomas (STS) and bone sarcomas (BS) patients, assessing both efficacy and toxicity profile.

**Methods:**

We retrospectively collected data of STS and BS patients treated with ciIFO (14 g/m^2^ as a 14‐day continuous infusion every 4 weeks) at our Institute from January 2013 to April 2023. The analysis included patients’ demographic and clinical characteristics, pathology details, treatment history, radiological response rates (RR), and toxicities per Common Terminology Criteria for Adverse Events (CTCAE) v5.

**Results:**

Ninety eight patients were included in the analysis. Median age at diagnosis was 49 years, 61% had previously received ifosfamide (IFO) and 83% anthracycline in earlier lines of treatment. Disease control rate (DCR) was 32%, with 9 PR and 22 stable disease (SD). Synovial sarcomas had the best (DCR) (60%). No statistically significant differences in DCR were seen according to sex, previous exposure to ifosfamide‐based CT, or ciIFO treatment line. mPFS was 3.0 months; mOS was 11.2 months. ciIFO was well tolerated, with an 11% discontinuation rate and no severe renal toxicity observed.

**Conclusions:**

ciIFO showed activity across different lines of therapy, achieving a DCR of 32%, and appeared better tolerated than standard‐dose IFO regimens, even in a cohort of heavily pretreated patients (18% had an Eastern Cooperative Oncology Group performance status (ECOG PS) ≥ 2). Synovial and bone sarcoma patients derived the greatest benefit (6‐month PFS of 45% in both groups).

## 1 Introduction

Soft tissue sarcomas (STS) and bone sarcomas (BS) are rare and heterogeneous neoplasms, comprising more than 80 different histological entities according to morphological and immunohistochemical features, specific biologic behavior and sensitivity to systemic treatments [1, 2, 3]. Multidisciplinary management in referral centers is recommended and requires close cooperation between sarcoma‐dedicated specialists. Unfortunately, in the metastatic setting, where a curative loco‐regional approach is excluded, systemic treatment choices are limited, and prognosis remains poor, with a median survival of 24 months in the largest series [4].

A single‐agent anthracycline represents a well‐recognized standard first‐line regimen in metastatic or advanced STS [5]. Combination regimens with anthracyclines and standard‐dose ifosfamide or dacarbazine, whose response rates do not exceed 30%–35%, are reserved for selected symptomatic patients where the highest possible response rate (RR) is needed [2, 5, 6]. Although some suggested sequences of therapies have been reported for individual histologies, second and further‐line regimens are not standardized, and the medical oncologist must evaluate the best balance between delaying disease progression and maintaining an acceptable quality of life for the patient [7]. Therefore, in the absence of clear indications on the treatment sequences, second or further‐line treatments are generally chosen based on histologies, on the patient's performance status and on the expected drug‐related toxicities. Common treatment options in the palliative setting include ifosfamide (IFO), trabectedin, pazopanib, gemcitabine ± docetaxel, dacarbazine, and eribulin, with response rates ranging from 0% to 15% [7, 8, 9].

Among these, IFO, an alkylating agent, has been used as a single agent in many different schedules of administration, the most common being high‐dose IFO (HDIFO), at 9–15 g/m^2^ continuous infusion over 3–5 days. This regimen has shown efficacy, especially in osteosarcoma, dedifferentiated chondrosarcoma, and synovial sarcoma, though with a heavy toxicity burden that includes myelosuppression, renal, neurological, and gastrointestinal (nausea and vomiting) toxicity [10, 11].

Continuous infusion IFO (ciIFO), consisting of 1 g/m^2^/day for 14 consecutive days every 4 weeks, represents an alternative option with activity even in those patients previously treated with the conventional combination of doxorubicin and IFO [11]. Administration is manageable and can achieve disease control in some histotypes [12]. However, despite its frequent use in clinical practice, data on its efficacy as second or further line in STS and BS are scarce. We present the results of a single‐center retrospective analysis of patients treated with ciIFO, assessing efficacy according to treatment line and histological subtype, as well as the toxicity profile.

## 2 Patients and Methods

A retrospective analysis of clinical and pathological data was conducted on STS and BS patients treated with ciIFO at Humanitas Research Hospital between January 2013 and April 2023. In our institutional practice, ciIFO is administered in the outpatient setting through a portable elastomeric pump containing 250 mL of normal saline (0.9% NaCl), delivering 1 g/m^2^/day of ifosfamide together with equimolar mesna (1 g/m^2^/day) for uroprotection. Each 250 mL elastomeric pump delivers continuous infusion for 7 days; on day 8, the device is replaced in the day‐hospital to continue treatment for another 7 days, completing the planned 14‐day continuous infusion. Patients are instructed to maintain adequate oral hydration (≥ 2.5 L/day), with intravenous fluids provided only in cases of renal impairment or poor oral intake. Neither primary G‐CSF nor antiemetic prophylaxis is routinely administered, but both are introduced in subsequent cycles if grade ≥ 3 toxicity occurs. Patients who received concomitant radiotherapy during ciIFO and those who were treated with ciIFO in a neoadjuvant/adjuvant setting were excluded from the analysis. The final follow‐up date was March 15, 2024.

Clinical and demographical data, including sex, age at diagnosis, previous treatments, outcomes, as well as pathological features (histological subtype, stage at diagnosis, extent of disease at baseline) were collected. Moreover, we gathered information on the ciIFO line, number of cycles, best response, and, where available, hematological, renal, and neurological toxicities, according to the National Cancer Institute (NCI) Common Terminology Criteria for Adverse Events (CTCAE) version 5 [13]. Data were collected in an anonymized, encrypted electronic database. The study was conducted according to the Declaration of Helsinki and received approval from our internal Ethics Committee.

### 2.1 Statistical Analysis

All evaluations were performed in the ITT (intention‐to‐treat) population, considering as evaluable all patients who received at least 1 day of treatment. Data were summarized as frequencies and proportions or as medians and ranges. Differences between study groups were assessed using the Chi‐square test for categorical data or Fisher's exact test when appropriate. Cox proportional hazards regression model was performed, and hazard ratios (HRs) were reported with 95% confidence intervals.

Progression‐free survival (PFS) and overall survival (OS) were analyzed using the Kaplan–Meier method, and differences between groups were evaluated with the log‐rank test. A *p*‐value ≤ 0.05 was considered statistically significant. All analyses were performed using SAS version 9.4.

## 3 Results

### 3.1 Patients’ Demographics and Disease Characteristics

Ninety‐eight adult patients with STS or BS were identified, including 42 females (43%) and 56 males (57%), with a median age at diagnosis of 48.5 years (range 15–81 years). The most common primary tumor sites were the extremities (40%), retroperitoneum (21%), and trunk (18%). Among the 87 patients with STS, the most frequent histological subtype was liposarcoma (25/87; 29%), followed by small round cell sarcomas (14/87; 16%). Eleven patients with bone sarcoma were included in the analysis, 7 of whom had osteosarcoma (7/11; 64%), and 4 had chondrosarcoma (4/11; 36%). Ewing sarcomas were classified as small round blue cell tumors, regardless of their primary anatomic site. Additional details are provided in Table 1 and Appendix A.

**TABLE 1:** Features of STSs and BSs patients treated with ciIFO.

Factor	*N* (%)
**Age**	49 years (15–81 years)
**Primary site** ^a^	
Extremity	39 (40%)
Retroperitoneum	21 (21%)
Trunk	18 (18%)
Other	20 (20%)
**Histological categories** ^a^	
Liposarcoma	25 (26%)
Small round cell sarcoma	14 (14%)
Bone sarcoma	11 (11%)
UPS/myxofibrosarcoma	13 (13%)
Synovial sarcoma	10 (10%)
Leiomyosarcoma	3 (3%)
Others	22 (22%)
**Stage at ciIFO start**	
Localized/locally advanced	16 (16%)
Distant metastases	82 (84%)
**Treatment line**	
1st line	20 (20%)
2nd line	50 (51%)
≥ 3rd line	28 (29%)
**Previous exposure to ifosfamide**	
IFO‐naïve	38 (39%)
Non‐IFO‐naïve	60 (61%)
**ECOG PS at ciIFO start** ^a^, ^b^	
0	37 (49%)
1	21 (28%)
≥ 2	18 (24%)

^a^ Percentages may not total 100 due to rounding.

^b^ Data missing on 22 patients.

Out of 98 patients, 20 (20%) received ciIFO as first‐line therapy. Among these, 17 had received prior perioperative chemotherapy (16 anthracycline‐based regimens, 1 IFO monotherapy regimen). Fifty patients (51%) received ciIFO in second‐line therapy, and 28 (29%) in further lines of treatment. At the start of ciIFO, 16/98 (16%) patients had localized or locally advanced disease, whereas 82/98 (84%) presented with distant metastases. Additionally, 59% of patients (58/98) had an Eastern Cooperative Oncology Group performance status (ECOG PS) of 0–1. Thirteen of 98 (13%) patients had an iatrogenic single kidney due to en‐bloc resection of the primary tumor, 60/98 (61%) had previously received IFO in perioperative or earlier lines of treatment, and 81/98 (83%) had undergone a prior doxorubicin‐based regimen.

The median number of ciIFO cycles administered was 3 (range 1–17); 11/98 patients (11%) received more than six cycles of therapy. At the time of last follow‐up, 78/98 patients (80%) had died; 18 patients were still living with disease, one was alive without macroscopic evidence of disease, and 1 was lost to follow‐up. Apart from the 11 patients who discontinued ciIFO due to treatment‐related toxicity (see Toxicity section), treatment interruption occurred because of disease progression in the vast majority of cases, with only a few exceptions (loss to follow‐up, surgery after SD, transition to maintenance therapy, or continuation of treatment at another institution).

### 3.2 Response

Radiological response to ciIFO was evaluable in 91/98 patients (93%); in the remaining 7 patients, radiological assessment was not feasible due to rapid clinical deterioration following just one cycle of treatment. All analyses were conducted on the ITT population of 98 patients, with the aforementioned 7 patients classified as having clinical progressive disease (PD).

Among the ITT population, we found a disease control rate (DCR) of 32% (31/98 pts), with 9 patients (9%) experiencing a partial response (PR), 22 patients (22%) achieving stable disease (SD), and 67 patients (68%) showing PD with ciIFO. DCR was similar between males and females, although with a higher incidence of partial response among females (Table 2). No significant differences in DCR were reported based on the ciIFO treatment line. Interestingly, we found a DCR of 37% (22/38) in the cohort of patients previously exposed to IFO, while for IFO‐naïve patients, the DCR was 24% (9/29), although the difference is not statistically significant (*p* = 0.19).

**TABLE 2:** Disease Control Rate and Best response to ciIFO in the ITT population, and according to sex, ciIFO treatment line, previous exposure to IFO and histological subcategories.

	DCR		Best response		
	%	*p*	PR (%)	SD (%)	PD (%)
**ITT Population** (N)	32%		9 (9%)	22 (22%)	67 (68%)
**Sex**		0.82			
Females (42)^a^	33%		7 (17%)	7 (17%)	28 (67%)
Males (56)^a^	30%		2 (4%)	15 (27%)	39 (70%)
**ciIFO treatment line**		0.81			
1st line (20)	35%		1 (5%)	6 (30%)	13 (65%)
2nd line (50)^a^	29%		5 (10%)	9 (18%)	35 (71%)
≥ 3rd line (28)	34%		3 (10%)	7 (24%)	19 (66%)
**Previous exposure to IFO**		0.19			
IFO‐naïve (38)	24%		4 (11%)	5 (13%)	29 (76%)
Non‐IFO‐naïve (60)^a^	37%		5 (8%)	17 (28%)	38 (63%)
**Histological categories**		0.45			
Liposarcoma (25)	24%		0 (0%)	6 (24%)	19 (76%)
Small round cell sarcoma (14)	21%		0 (0%)	3 (21%)	11 (79%)
Synovial sarcoma (10)	60%		1 (10%)	5 (50%)	4 (40%)
Bone sarcoma (11)	36%		2 (18%)	2 (18%)	7 (64%)
UPS/myxofibrosarcoma (13)	38%		2 (15%)	3 (23%)	8 (62%)
Leiomyosarcoma (3)	33%		1 (33%)	0 (0%)	2 (67%)
Others (22)^a^	27%		3 (14%)	3 (14%)	16 (73%)

^a^ Percentages may not total 100 due to rounding.

Among the different histological subcategories, synovial sarcomas (SS) had the highest DCR of 60% (1 PR + 5 SD), while DCR was 33%, 24%, 38%, 21%, 36%, and 27% for leiomyosarcoma (LMS), liposarcoma (LPS), myxofibrosarcoma and undifferentiated pleomorphic sarcoma (UPS), small round cell sarcoma (SRCS), BS and others, respectively (*p* = 0.45) (Table 2).

### 3.3 Survival

At the time of the analysis, the median follow‐up was 39.2 months (range 0.4–100.2).

In the whole population, the median progression‐free survival (mPFS) was 3.0 months. Three‐month and 6‐month PFS were 51% and 24%, respectively. The median overall survival (mOS) was 11.2 months. One‐year and 2‐year OS were 48% and 30%, respectively.

**FIGURE 1:**
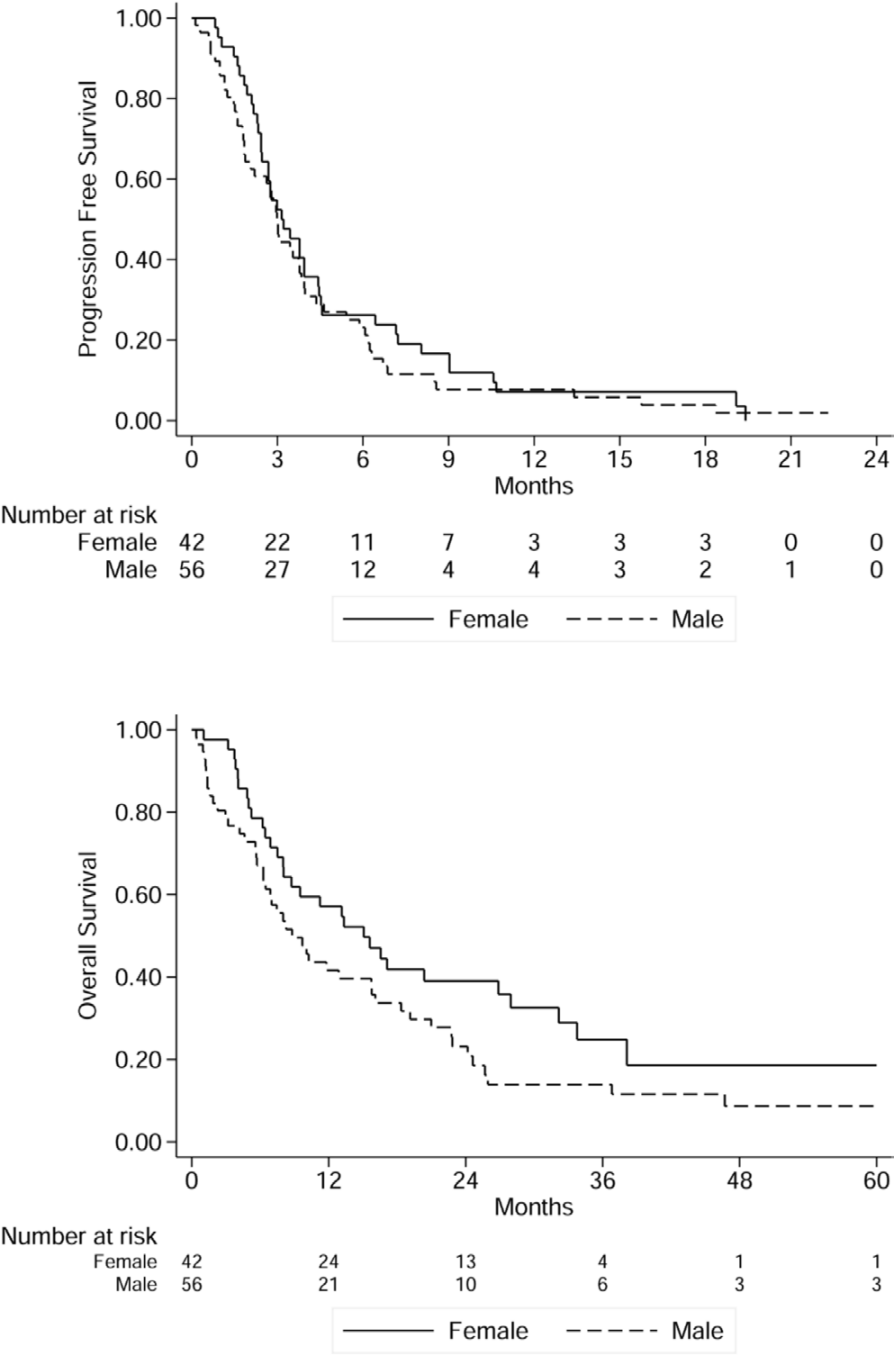
Progression free survival and overall survival by sex: (a) mPFS by sex, (b) mOS by sex.

There was no statistically significant difference in terms of PFS by sex: mPFS was 3.2 and 3.0 months in females and males, respectively (*p* = 0.35). Regarding OS, females had slightly better mOS compared to males, 15.1 months versus 8.8 months, although still not statistically significant (*p* = 0.07) (Figure 1a,b). Similarly, no statistically significant differences in PFS and OS were observed according to ciIFO treatment line and previous exposure to IFO. As expected, patients with non‐operable locally advanced disease had a statistically significant longer OS compared to metastatic patients, with mOS of 15.6 months and 6.4 months, respectively (*p* < 0.01), while mPFS did not differ significantly in the two groups.

Regarding histological subtypes, no statistically significant difference was observed for PFS and OS among different groups. SS and BS had the best survival rates, with a mPFS of 3.9 months and 4.0 months, 90% and 81% of patients alive and progression free 3 months after starting ciIFO, and a median OS of 21.0 months and 16.5 months, respectively.

**FIGURE 2:**
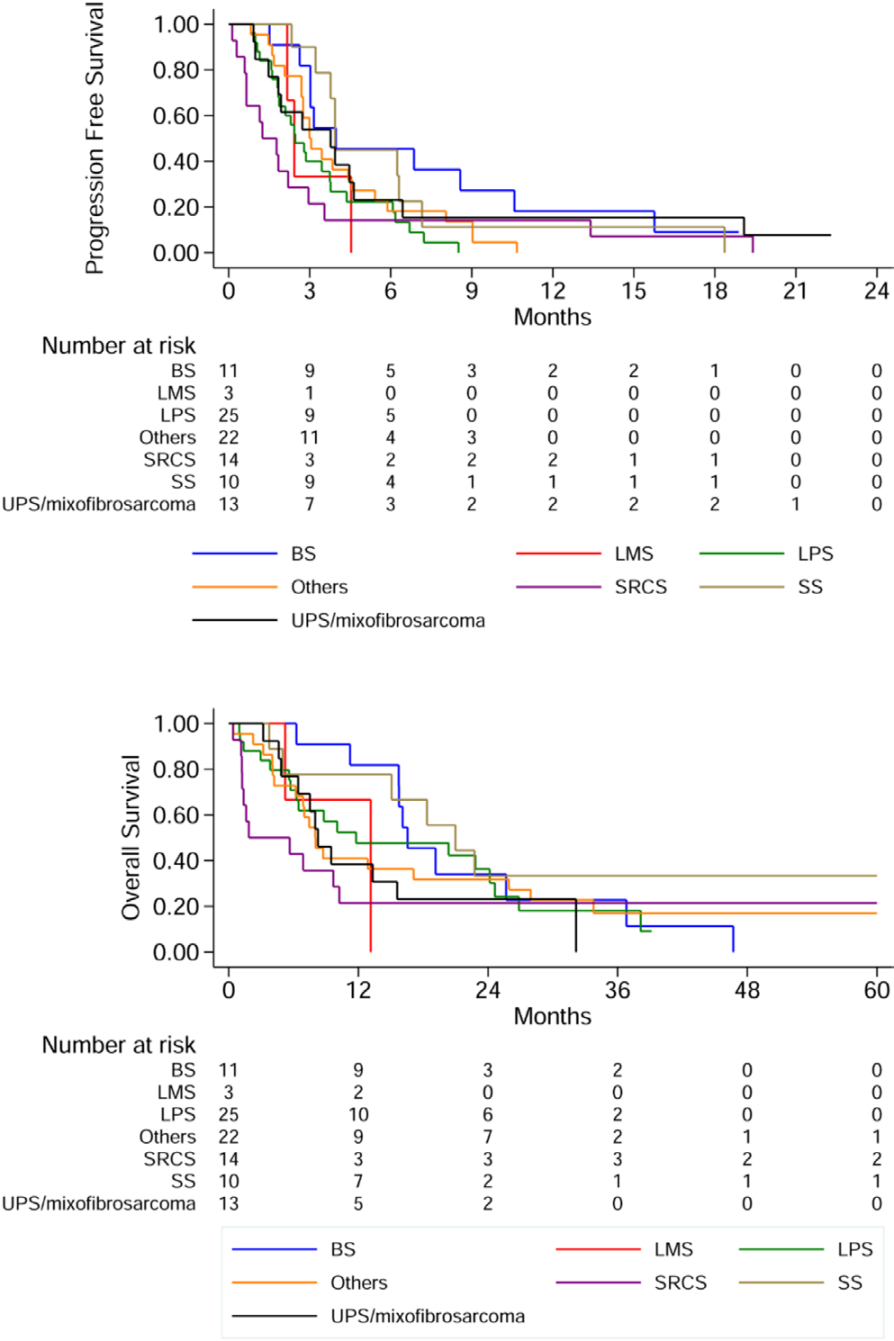
(a) Progression free survival and (b) overall survival by histological subcategory.

In the multivariable Cox regression model including sex and metastatic stage, the trend toward improved OS for females persisted. Although not statistically significant, the estimated effect suggests a potentially relevant signal, with females showing a lower risk of death compared to males (HR 0.65, 95% CI, 0.41–1.03, *p* = 0.064). No meaningful differences by sex were observed for PFS. Metastatic stage remained the strongest predictor of outcome, with nonmetastatic patients experiencing a substantially lower risk of death (HR 0.39, 95% CI, 0.22–0.68, *p* = 0.001). Additional survival data is reported in Tables 3 and 4, and Figure 2a,b.

**TABLE 3:** Median PFS and OS in the ITT population, and according to sex, ciIFO treatment line, previous exposure to IFO, stage at ciIFO start, and histological subcategories.

	mPFS (months)	95% CI	*p*	mOS (months)	95% CI	*p*
**ITT Population**	3.0	2.7–3.8		11.2	8.0–16.1	
Sex			0.35			0.07
Females	3.2	2.5–3.9		15.1	8.1–28.0	
Males	3.0	2.1–3.8		8.8	6.3–15.8	
**ciIFO treatment line**			0.14			0.65
1st line	3.5	2.2–4.4		9.7	5.7–19.2	
2nd line	3.1	2.5–3.9		15.6	7.0–24.6	
≥ 3rd line	2.8	1.6–3.8		10.1	5.7–20.4	
**Previous exposure to IFO**			0.98			0.91
IFO‐naïve	3.0	2.4–4.0		13.1	6.3–20.4	
Non‐IFO‐naïve	3.0	2.1–3.8		10.1	7.5–16.6	
**Stage**			0.25			< 0.01
Non‐metastatic	3.0	2.6–3.8		15.6	8.3–21.0	
Metastatic	3.4	1.3–4.0		6.4	1.4–13.2	
**Histological categories**			0.15			0.80
Liposarcoma	2.5	1.8–3.7		11.8	5.7–24.6	
Small round cell sarcoma	1.5	0.6–3.0		3.8	1.2–10.3	
Synovial sarcoma	3.9	2.3–7.2		21.0	3.7‐n.r.	
Bone sarcoma	4.0	2.6–10.6		16.6	11.2–36.8	
UPS/myxofibrosarcoma	3.8	1.5–4.6		8.3	4.9–15.6	
Leiomyosarcoma	2.4	2.2–4.5		13.1	5.2–13.1	
Others	3.0	2.7–4.6		8.0	4.2–25.9	

**TABLE 4:** Progression‐free and overall survival rates according to histological subcategories.

Histological categories	PFS		OS	
	3‐month	6‐month	6‐month	1‐year
Liposarcoma	40%	22%	71%	48%
Small round cell sarcoma	21%	14%	43%	21%
Synovial sarcoma	**90%**	**45%**	78%	78%
Bone sarcoma	**82%**	**45%**	100%	82%
UPS/myxofibrosarcoma	53%	23%	77%	38%
Leiomyosarcoma	33%	0%	67%	67%
Others	50%	18%	73%	41%

*Note:* The bold was to highlight the highest 3‐ and 6‐month PFS reached in synovial and bone sarcomas.

### 3.4 Toxicity

ciIFO treatment was generally well tolerated. Overall, the most common adverse events were hematological. Thirty‐six out of 98 patients (37%) experienced any‐grade hematological toxicity, of whom 16 (16%) experienced severe events (grade ≥ 3 per CTCAE). Four cases of renal toxicity were reported (4%), all non‐severe. Among these 4 patients, only one had a single kidney due to en‐bloc resection of the primary retroperitoneal tumor. Among the other monokidney patients (N = 13), four experienced hematological toxicity (2 grade 2; 2 grade 4) and three permanently discontinued treatment due to a treatment‐related adverse event (AEs): G3 thrombocytopenia, G4 neutropenia, and G3 mucosal toxicity.

Two cases of neurological toxicity (visual hallucinations, confusion) were reported, one occurring after the first ciIFO cycle and the other occurring after the third cycle. In both cases, neurological symptoms resolved after the interruption of the infusion, and re‐administration of the drug was feasible with adequate supportive care.

Overall, severe (e.g., G ≥ 3) AEs occurred in 20/98 patients (20%): 16 hematological, 2 upper gastrointestinal (e.g., nausea and vomiting), 1 mucosal, and 1 allergic reaction. The discontinuation rate due to treatment‐related AEs was 11%. One patient permanently discontinued treatment due to an allergic reaction after the 2nd cycle, which manifested as a diffuse pruritic erythema on the limbs and trunk. Further details on AEs leading to treatment discontinuation are provided in Table 5.

**TABLE 5:** Adverse events to HDIFO conditioning the interruption of the treatment.

Patient (*N*°)	Cause of interruption	*N*° of cycles	Best response	Subsequent treatment
#11	Nausea and vomiting	3	PD	Yes (pazopanib)
#32	G3 hemorrhoids	1	PD	Yes (eribulin)
#37	G3 low platelet count	1	PD	Yes (etoposide, trabectedin, gemcitabine)
#51	G3 low WBC count	1	PD	No
#63	Subjective intolerance	1	SD	No
#75	Nausea and vomiting	1	PD	No
#78	G3 anemia	2	PD	Yes (gemcitabine)
#81	Allergic reaction	2	PD	Yes (doxorubicin)
#90	Subjective intolerance	1	PD	No
#97	Hallucinations/confusion	3	PD	No
#101	G3 low platelet count	1	PD	No

## 4 Discussion

This retrospective single‐center analysis exploring the role of ciIFO on an adult population of locally advanced or metastatic STS and BS patients confirms the efficacy and safety of ciIFO in this setting. We found a DCR of 32% and a PR of 9% across the entire population regardless of the line of treatment. In the cohort of patients previously treated with standard‐dose IFO, we observed a DCR of approximately 37% regardless of the time since discontinuation of the prior regimen. Moreover, ciIFO exhibited a well‐manageable toxicity profile, with only 11 out of 98 patients discontinuing therapy due to treatment‐related adverse events.

Ifosfamide has long been established as an active treatment for advanced sarcoma [10, 11, 14]. It has primarily been studied in combination with anthracyclines, exploring various dosing and scheduling strategies [15, 16, 17]. Currently, the combination of ifosfamide and anthracyclines (AI) represents the standard treatment in the perioperative setting (neoadjuvant and adjuvant chemotherapy) [2, 18]. A study investigating the non‐inferiority of histotype‐tailored neoadjuvant chemotherapy compared to AI has failed across all histologies except for myxoid liposarcoma; in this study, ifosfamide was selected as the treatment for synovial sarcoma due to its well‐established efficacy in this histological subtype [19].

Moving to the inoperable or metastatic STS setting, anthracycline monotherapy remains the standard treatment, supported by a large phase III trial comparing AI to doxorubicin alone [5]. The trial showed superior PFS and RR for the combination, but no difference in OS, along with a significantly higher toxicity (nearly 50% of patients experienced G3–4 hematological toxicity). As a result, monotherapy is preferred, while the combination of anthracycline and ifosfamide is used when tumor shrinkage is needed (e.g., due to symptomatic disease).

Ifosfamide monotherapy is currently used as second‐line or later treatment, even in patients previously treated with the combination. A phase III trial evaluated two different schedules of ifosfamide (a continuous infusion of 9 g/m^2^ over 3 days versus a shorter infusion of 3 g/m^2^ per day over 3 h for 3 days), finding no significant differences in PFS and OS between the two arms [17]. However, the continuous infusion arm demonstrated a higher RR compared to the short infusion (8.4% vs. 5.5%, respectively) [17]. Since a dose‐efficacy relationship has been documented [20], high dose regimens (HDIFO, 9–15 g/m^2^/cycle) have been developed particularly for BS, achieving up to a 40% response rate [11, 21]. However, dose escalation results in a high incidence of serious adverse effects (hematologic, renal, and neurologic toxicity) [22]. To improve the toxicity profile, continuous infusion schedules have been implemented both in STS and BS, initially as a 3–5‐day infusion [8, 23] and later as a prolonged 14‐day infusion [12, 24, 25], allowing for reduced toxicity while maintaining efficacy, as described in our study.

When analyzing histology‐specific efficacy, SS has been described as highly sensitive to IFO, in particular based on the data of some small series of patients [10, 26, 27]. However, other studies have reported contrasting evidence: the study by Spurrel et al. of 25 SS patients reported a 25% RR for IFO monotherapy [28], and the retrospective analysis by Sleijfer et al. showed equivalent OS, PFS, and RR for SS patients treated with doxorubicin compared to IFO‐based therapies [29]. We observed significantly higher efficacy in SS compared to other histologies (DCR of 60%), therefore supporting the thesis of SS sensitivity to ifosfamide [30]. Reasons for this discrepancy could be related to the fact that these studies were conducted before the times of central pathology review, so probably harboring cases of misdiagnosed histology. On the other hand, while a higher DCR (up to approximately 71%), has been described in the past for dedifferentiated liposarcoma (DDLPS) [12], in our study only 3/17 DDLPS patients (18%) had SD, and no PR were observed. Perhaps this difference might be explained by the fact that our DDLPS patients were heavily pretreated: 6/17 patients had received perioperative chemotherapy, 11/17 received ciIFO in 2nd line, and 4/17 in 3rd line.

Even though STS represented the majority of our sample, we also included high grade BS (osteo and chondrosarcoma, *N* = 11). We confirmed that ciIFO has relevant clinical activity with a DCR of 36% (4/11) and a 6‐month PFS of 45%, showing efficacy and good tolerance as reported in a recent similar retrospective cohort study in BS [25]. Interestingly, the metronomic modality (14 days infusion) shows a similar effect to 5 days infusion, suggesting this regimen as the optimal schedule even if there are no randomized trials.

The multivariable analysis confirmed that sex was not a statistically significant predictor of survival outcomes, yet the hazard ratio for OS suggests a noteworthy tendency favoring female patients. Although the *p* value of 0.064 does not meet the conventional significance cutoff, it remains close enough to indicate a possible underlying effect, consistent with the observation that the OS curve for females stayed above that of males throughout follow up. While this finding is not conclusive, it may be of interest for future investigations focused on sex‐based differences, particularly given the growing relevance of gender considerations in oncology research.

In the entire ITT population, we found a mPFS of 3.0 months, with 3‐month and 6‐month PFS rates of 51% and 24%, respectively. When compared to the data reported by Van Glabbeke et al. [31], our findings suggest that ciIFO represents an active second‐line treatment for sarcoma patients. Specifically, in the Van Glabbeke study, a 3‐month and 6‐month progression‐free rate (PFR) of ≥ 40% and ≥ 14% respectively was considered indicative of drug activity for second‐line therapy. Since our results overcame both thresholds, we confirmed the therapeutic validity of ciIFO. However, in our study as in Van Glabbeke's, the treatment response varied by histological subtype, underscoring the importance of considering both the line of treatment and the histological subtype when evaluating therapeutic strategies for sarcoma.

In our study, neither PFS nor OS was significantly different between IFO‐naïve patients and the ones that had previously received ifosfamide. This finding supports the hypothesis of drug‐resistance circumvention through dose intensification [10, 32].

Efficacy of ciIFO is comparable with other second or further line treatments for STS. Among chemotherapy regimens that have been evaluated in this setting, gemcitabine (+/− docetaxel) and dacarbazine have conferred similar results (mPFS 3–4.4 months) [33, 34, 35, 36]. Analogously, trabectedin has shown efficacy in STSs, granting a significant improvement of mPFS in STS over dacarbazine (4.2 months vs. 1.5 months) [36]. Based on these data, the choice of second/further line therapy still depends on histology and patient characteristics. Comparative prospective studies are needed to better define and individualize the best treatment sequence for patients with sarcoma.

Besides efficacy, safety and comfort of the patient play a crucial role in advanced disease. In our study we documented no treatment‐related deaths, and very few serious treatment‐related adverse events leading to treatment discontinuation (11%). Grade 3 or higher AEs occurred in 20% of patients and were mainly hematologic toxicity that reversed after discontinuation. Even though 13% of patients in our sample had post‐operative single‐kidney, no patients experienced renal impairment during ciIFO treatment. Moreover, ciIFO can be carried on as an outpatient setting, not requiring hospitalization. These data show the good tolerability of ciIFO in real‐world practice, offering a treatment option that allows to maintain a good quality of life even in a population with a significant proportion of patients with ECOG PS ≥ 2. Given the DCR observed in BS (36%), this regimen could represent a valuable “metronomic” option, particularly for BS patients with impaired renal function or frail individuals who may not tolerate HDIFO.

Our study has several limitations, including its retrospective study design and the heterogeneity of the patient sample in terms of histology, extent of disease, and treatment sequences. In line with contemporary sarcoma oncology, we explored treatment activity by histological subtype (Appendix A). However, an important limitation of this histology‐driven analysis is that several subtypes were represented by extremely small numbers of patients, in some cases a single individual. Another limitation is the difficulty in providing a standardized summary of dose modifications and delays, as ciIFO dosing in real‐world metastatic sarcoma practice is often individualized from the outset, leading to heterogeneous schedules and adjustments that cannot be reliably aggregated.

Despite these limitations, our analysis has several strengths. A key strength is the multidisciplinary management of each patient within a sarcoma‐dedicated tumor board, including centralized pathologic review of each histologic sample by an internal team of expert sarcoma pathologists. Furthermore, our analysis helps contextualize the role of ciIFO among the therapeutic options for patients with advanced sarcoma in real‐world practice. In this context, while histology remains central to treatment selection, the favorable tolerability of ciIFO across multiple lines supports its consideration as a therapeutic option in selected patients with advanced disease, particularly when alternative treatments are limited.

## 5 Conclusion

Continuous infusion Ifosfamide (ciIFO) represents a well‐tolerable and manageable chemotherapy regimen, offering a valuable therapeutic option for second or further‐line treatment for metastatic patients with sarcoma, including those pretreated with IFO, frail patients, or those with a post‐operative single kidney. Treatment decisions should be individualized, integrating histological subtype, clinical features, patient preferences, and available therapeutic options. Our observations underscore ciIFO's versatility and potential as a therapeutic option in several clinical scenarios, contributing to a comprehensive management of sarcoma patients, particularly those with bone sarcomas and synovial sarcomas. Finally, our results provide valuable reference data for further clinical trials exploring ciIFO as a second‐line therapy for sarcoma.

## Author Contributions

Conceptualization: I.M., A.F.B., A.L., M.S.G., R.G.M.; Methodology: A.F.B., A.L., L.G., M.S.G., R.G.M.; Formal analysis: I.M., L.G.; Investigation: I.M., S.F., E.S., M.L.M., M.S.G.; Writing – original draft: I.M.; Writing – Review and Editing: I.M., A.L., M.S.G., F.C.M.C., L.S., S.L.R., F.D., P.N., E.C., C.C., R.G.M. A.S., A.F.B.; Visualization: I.M., M.S.G., L.G.; Supervision: R.G.M., A.F.B., A.S. All authors have read and approved the final version of the manuscript. All authors have agreed to be accountable for all aspects of the work in ensuring that questions related to the accuracy or integrity of any part of the work are appropriately investigated and resolved.

## Funding

The authors have nothing to report. The publication fee for this work was covered by the Italian Ministry of Health’s “Ricerca Corrente” funding to the IRCCS Humanitas Research Hospital.

## Ethics Statement

The study (Protocol Code: ONC/OSS‐19/2023) received administrative acknowledgment from the local Ethics Committee (Reference No. 30/23, dated September 19, 2023), in accordance with Italian regulations on the protection of personal data.

## Consent

This was a retrospective and observational study with no impact on patient management; therefore, informed consent was not required, as per the ethics committee's acknowledgment. All patients signed the institutional privacy policy for the processing of personal data. All aspects of this study were conducted in accordance with the principles of the Declaration of Helsinki.

## Conflicts of Interest

The authors declare no conflicts of interest.

## Data Availability

The data that support the findings of this study are available from the corresponding author upon reasonable request.

## Appendix A

### Histological subcategories and corresponding best response

**Table:**

Histological category	Histologies	*N*	Best response		
			PR	SD	PD
Liposarcoma (*N* = 25)	Dedifferentiated liposarcoma	17		3	14
	Myxoid liposarcoma	8		3	5
Small round cell sarcoma (*N* = 14)	DSRCT	2		1	1
	Alveolar rhabdomyosarcoma	1			1
	Embryonal rhabdomyosarcoma	1		1	
	CIC‐rearranged sarcoma (Ewing‐like)	1			1
	Extraskeletal Ewing sarcoma	8		1	7
	Ewing sarcoma of the bone	1			1
Synovial sarcoma (*N* = 10)	Synovial sarcoma	10	1	5	4
Bone sarcoma (*N* = 11)	Chondrosarcoma	3	1		2
	Mesenchimal chondrosarcoma	1		1	
	Osteosarcoma	7	1	1	5
UPS/myxofibrosarcoma (*N* = 13)	Myxofibrosarcoma	4		1	3
	Pleomorphic sarcoma	2	2		
	UPS	7		2	5
Leiomyosarcoma (*N* = 3)	Leiomyosarcoma G2	1	1		
	Leiomyosarcoma G3	2			2
Others (*N* = 22)	Angiosarcoma	1	1		
	Mixoid extraskeletal chondrosarcoma	2		2	
	Epithelioid hemangioendothelioma	1			1
	Inflammatory pleomorphic malignant histiocytoma	1			1
	Low‐grade mesenchymal neoplasm	1			1
	Pleomorphic rhabdomyosarcoma	1			1
	Epithelioid sarcoma	2		1	1
	MDM2 amplified intimal sarcoma	1			1
	MPNST sarcoma	2			2
	NOS sarcoma	2			2
	Endometrial stromal sarcoma	4			4
	Solitary fibrous tumor	4	2		2
